# Gender and Pain in Nineteenth‐Century Cancer Care

**DOI:** 10.1111/1468-0424.12468

**Published:** 2020-03-05

**Authors:** Agnes Arnold‐Forster

In the 1850s, an American surgeon Dr J. Weldon Fell appeared in London claiming to possess a new cure for cancer. Soon after his arrival in the metropolis in 1856, he applied to the Middlesex Hospital's cancer ward to trial his treatments on the patients held within. The ward had, since its foundation in 1792, adopted the unusual policy of ‘rendering [itself] available for the trial of every new method of treatment which could with safety and propriety be adopted’.^1^ The surgical staff, ‘ever alive to the importance of doing all in its power to advance the treatment of this intractable complaint’, agreed and wrote up their assessment of his ‘plan’ in their minutes before publishing it as a separate volume. This article will look at this ‘plan’ alongside another account of Fell's treatment, written by the naturalist Philip Henry Gosse about his wife, Emily Bowes Gosse.^2^ Both accounts record intense physical and emotional suffering, and this article will explore what purpose these textured narratives of pain served and what they can reveal about the dynamics of Victorian masculinity in the context of incurable disease.

Both the *Report of the Surgical Staff of the Middlesex Hospital* and Gosse's account, *A Memorial of the Last Days on Earth of Emily Gosse*, were published in 1857. The former was produced by the prominent English medical publisher, John Churchill, and the latter by James Nisbet & Co., of Berners Street, London. Fell's treatment and the surgical staff's report were discussed widely in the medical press and *The British Medical Journal* reviewed the report positively: ‘The result of their labours reflects on them great credit. It is remarkable for its candour, and offers a most satisfactory justification’.^3^ Gosse's account is a slim, pocketbook‐size volume that was ‘at first intended…only for private circulation among friends’ and only five copies are thought to exist.^4^ However, while he claimed that the ‘simple record’ might just be useful ‘for the stirring up of the faith and love’ of those who did not know his wife, the tone and content of the account suggest that he intended for it to be read by people with some degree of medical knowledge.^5^

In what follows, I will argue that the surgical staff of the Middlesex Hospital used their patients’ descriptions of agony, disgust and incapacity to assess the therapeutic efficacy of Fell's treatment. This process was particularly important because the surgical staff believed Fell's intervention to be palliative rather than curative and so could not rely on observable, clinical signs of therapeutic success or failure. Instead, doctors had to depend on a complex calculus of pain and suffering and consider an expansive and holistic interpretation of health and wellbeing to decide whether to incorporate Fell's treatment into their clinical arsenal. I will also suggest that both the hospital's surgical staff and Philip Henry Gosse used their affective narratives of female suffering to construct an image of caring and invested professional men; in doing so, I argue, they subverted or complicated traditional ideas of Victorian masculinity and scientific detachment. This article, therefore, supports the insistence in masculinity studies that it is more accurate to speak of a plurality of masculinities rather than a stable, hegemonic singularity. John Tosh argues that ‘the dominant code of Victorian manliness’ emphasised self‐control, stoicism, hard work and independence.^6^ However, that identity shifted across the period and Stefan Collini calls for a ‘more diverse, flexible and just plain ragged’ conceptualisation of normative masculinity in the nineteenth century.^7^

Indeed, the practitioners’ interest in the emotional state of their patients runs counter to conventional ideas of the nineteenth‐century surgeon – who has been portrayed by historians as detached, uninterested or even sadistic.^8^ Similarly, Gosse's highly emotional account of his wife's death and dying cuts across our expectations of Victorian masculinity and scientific detachment. Both the surgical staff and Philip Henry Gosse married medical and scientific skill and interest with compassion and care. Gosse was a naturalist, populariser of science and a Plymouth Brethren. In 1848 he married Emily Bowes, a forty‐one‐year‐old member of the Brethren and in 1849 she gave birth to their only son, Edmund.^9^ Much has been written about Gosse as a naturalist, theologian and the ‘father’ in the Edwardian memoir *Father and Son* (published by Edmund in 1907) in which his son described him as unloving and oppressive.^10^ With respect to his professional characteristics, historian Aileen Fyfe writes that Gosse, ‘won himself a reputation for observational accuracy and election to the Royal Society’^11^, and Michael Newton suggests that his ‘writings reveal a genuinely sweet character’.^12^ However, his account of his wife's death and dying has been subjected to less historical scholarship and has not been considered in the context of his scientific, masculine identity, or against the backdrop of mid‐nineteenth‐century cancer theory and practice.^13^

This is partly because the history of pre‐modern cancer remains understudied. While some scholars have made inroads into medieval, early modern and nineteenth‐century Britain, far more have traversed the disease's twentieth‐century terrain. This asymmetry can be partly explained by how studies of cancer and chronic disease have been constrained by a version of periodisation that serves to tie certain maladies – or malady‐types – to specific epochs.^14^ Recently, however, some historians have turned their attention to malignancy in nineteenth‐century Britain and considered the gendered nature of the disease in that period. For example, in her book and article, both on cancer and gender between 1860 and the 1940s, Ornella Moscucci explores the construction of cancer as a peculiarly ‘female’ disease.^15^ She draws on the arguments of historical sociologist Tammy Duerden Comeau who claims that early‐nineteenth‐century cancer was defined according to its supposedly ‘female’ characteristics – for example, she argues that its capacity for growth and extension was conceptualised as reproductive and generative.^16^ However, neither scholar addressed the role gender played in discussions of therapeutic efficacy and professional identity, or looked at the gendered expressions of pain and suffering in the cancer clinic.

This lacuna reflects a broader asymmetry in the history of nineteenth‐century science and medicine. While historians of masculinity have produced innovative work exploring the formation of masculine identities in many areas of life in Victorian Britain, as Heather Ellis argues, the ‘world of science has remained curiously unexamined’.^17^ Before the publication of Ellis's book, *Masculinity and Science in Britain, 1831–1918* in 2017, relatively little attention was paid to male self‐fashioning and, instead, gender historians focused on the exclusion of women from scientific cultures and knowledge‐making. An exception is Jan Golinsky, who argues that the archetypical scientist was ‘associated with distinctly masculine character traits, whether he is a man of action or cool rationalist, benevolent patriarch or glamorous young hero, saint or devil’.^18^ Ellis critiques the tendency of some historians to reify the male scientist in nineteenth‐century Britain as ‘a completely secure masculine persona, in control of discourse performance, structures, languages and theatres of power’.^19^ However, and as Golinsky noted, scientific masculinity was a construction and was, therefore, inherently unstable and vulnerable to attack.^20^ Michel Foucault argued that ‘care of the self’ was crucial to the construction of male scientific authority and gestured towards the centrality of the ‘preparatory exercises of self‐preservation’ that worked to present a coherent and powerful image of the masculine scientists to critics and detractors.^21^ For example, the chemist Humphry Davy was often accused of effeminacy and critics associated his scientific interests with a form of ‘diminished masculinity’.^22^

Ellis and Golinsky present a range of different ‘ideals of the man of science’. He could be a fashionable gentleman, effeminate, humble, moral, civically minded and lacking in empathy. Here, I add another image and ideal: the compassionate and affective scientist and surgeon. This article thus dialogues with a recent body of historical scholarship that explores the place of pain and suffering in nineteenth‐century surgery.^23^ Historian Lynda Payne argues that by the end of the eighteenth century a culture of emotional detachment suffused operative surgery.^24^ Various historians – both popular and academic – have reconfigured this detachment into insensitivity and dispassion and concentrated on the gendered nature of the relationship.^25^ The nineteenth‐century surgeon, immune to the suffering of his patient, is a familiar caricature. Building on arguments made by Joanna Bourke and Michael Brown, this article will suggest that this conceptualisation is unsatisfying and leaves out the many and various ways that mid‐nineteenth‐century practitioners made use of ‘compassion and intersubjectivity’ in the formation of their culture and identity.^26^

This article will begin by looking at cancer and its care in the mid‐nineteenth century before moving on to outline the nature and scope of Fell's ‘trial’. Then, I will explore the gendered nature of the ‘calculus of suffering’ practitioners deployed to assess the therapeutic efficacy of the treatment.^27^ In the second part of the article, I will look at Fell's care of Emily Bowes Gosse and her husband's account of that process. In both cases, I will consider the surgical staff of the Middlesex Hospital and Philip Henry Gosse, and interrogate their use of female suffering in their constructions of scientific masculinity and professional identity. Finally, and in line with the themes of this forum, I will show how male surgeons positioned themselves as expert analysers of their female patients’ pain and how gendered attitudes towards the suffering experienced by individual women are inextricable from broader ideas about social class.

## Cancer and its care in the mid‐nineteenth century

In November 1791, Mr. Howard of Argyll Street appeared at the Middlesex Hospital's weekly board meeting ‘and read a Paper on the Subject of Cancers’. He informed the governors ‘that a Friend proposed a contribution of three Thousand Pounds for establishing a Fund for the endowment of a Ward for the reception of Persons afflicted with that disorder, and four Hundred Pounds to fit up the Ward provided they should agree to the Plan contained in that Paper’.^28^ The board accepted, and Howard's paper was reproduced in full in the hospital's minutes. He wrote, ‘I take the liberty to observe that two principal objects present themselves to my Mind on this occasion…the relief of Persons afflicted with Cancer, and the Investigation of a Complaint’.^29^ In doing so he encapsulated the two explicit aims of the board: to provide care, palliative or otherwise, for cancer patients and to research the disease. Howard cited the ‘deplorable situation of cancerous Paupers’, and he called the disease ‘extremely common’ albeit ‘both with regard to its natural history, and cure, but imperfectly known’.^30^

Howard outlined a detailed plan for how the ward would be set up and managed. To relieve ‘persons afflicted with Cancer’ he suggested ‘that an airy Ward of the Middlesex Hospital might be appropriated to this specific Disease and to this Disease only, that the diseased might there find such alleviation of their sufferings as their respective situations should require, and that for an unlimited time’.^31^ He proposed that the ward be divided into two sections, one side for men and the other for women, ‘containing ten or twelve Beds…[and] the usual function of…bowls, candles, board’. They predicted that there would be about 40 in‐patients per year.^32^ The investigation of the disease was equally as important to Howard as was the care of its sufferers. As part of this research, Howard advocated the keeping of a detailed case history for every patient admitted: ‘In order to improve a subject…I propose, that a faithful account of the history and circumstances of every case be kept, its antecedents and consequences should be marked, the effects of medicines and of Operations, when necessary, noted, together with all the collateral details’.^33^ Howard believed that ‘much useful knowledge may be disseminated, and that we may in no great length of time be furnished with documents on the Disease and its cure, much more authentic than any we are at this time in possession of’.^34^

While the Middlesex treated both men and women, most of the patients cared for in the hospital were women. As suggested in the introduction, gender has been a key category of analysis for historians of cancer. The prominence of breast cancer in contemporary culture and society has made the disease a fertile ground for historians of medicine, both popular and academic. Frances Burney's account of her 1811 mastectomy has proven particularly alluring for feminist critiques of the medical gaze, and investigations into her words and experience form a substantial part of our knowledge and understanding of nineteenth‐century cancer.^35^ Most cancer patients in the nineteenth century were women – a fact observed and problematised by contemporaries – and their over‐representation was partly because their tumours more often appeared in easily accessible and visible organs: breasts and genitals. Thus, while men were treated at the Middlesex and encountered Fell's ‘cure’, women and their bodies are prominent in this article and most of the clinical interactions I interrogate took place between a male surgeon and his female patients.

Gender was not, however, the only category that structured the ‘cancer experience’ in the nineteenth century. The men and women who sought care at the Middlesex mostly walked the short distances from the surrounding areas of intense urban poverty. The ward was intended for the ‘cancerous pauper’, and across the period most hospital patients were drawn from the lower orders as wealthier Londoners preferred to pay doctors to attend to them in the comfort of their own homes. Most of the women examined in this article were, therefore, doubly disempowered – their vulnerability exacerbated by both their gender and their class. In contrast, Emily Bowes Gosse was educated, middle class and married to a teacher, naturalist and populariser of science. Unlike the patients of the Middlesex Hospital, she could afford personal care for her cancer and visited the surgeon in his own home.

## Dr J. Weldon Fell and his trial

The Middlesex's commitment to scientific investigation alongside the provision of care prompted the surgical staff to regularly invest in the appraisal of new and innovative treatments for cancer. Throughout the century, the ward received letters from people seeking the hospital's legitimising expertise and promising new cures. Fell was but one of many. In 1817, a ‘gentleman’ named Ashby applied to the Middlesex to trial his ‘remedy for cancer’ on the housed patients, ‘under the inspection of the medical officers’.^36^ Following their investigation, medical officers rejected Mr. Ashby's treatment, but this early failure did little to dampen their successors’ enthusiasm for experimentation. Thus, when Fell arrived in London in 1856, the Middlesex readily accepted his trial and were roundly praised in the press for their enlightened and ‘scientific’ approach to the ‘cancer problem’.^37^ The report produced by the surgical staff – containing lengthy and detailed case histories for each patient plus a narrative assessment of Fell's innovation – provides us with unusual insight into the clinical experience of poor women in the mid‐nineteenth century.

However, I do not want to suggest that this document gives us unmediated access to these women's thoughts and feelings. Their words were rendered by the surgeons who took their histories, listened to their complaints and interpreted their emotional and physical responses. Moreover, while the text is replete with ‘quotations’ from patients undergoing Fell's treatment, the practitioners had control over the words retained in the archive, and we have no way of knowing if they are accurate. The hospital is also an unusual environment, with strict gendered and classed hierarchies of power, and we can only speculate as to whether the female patients reported their true feelings to the male doctors. Nonetheless, I think we can derive a version of events and emotions from the surgical staff's report and disentangle some of the complex feelings and sensations that accompanied Fell's application. The report contains a diversity of patient responses – both positive and negative – and the accounts are often idiosyncratic and filled with individual personality. Finally, we can also determine that practitioners considered the patient voice valuable in their assessment process – this reveals something about intersubjectivity and the doctor‐patient relationship, irrespective of the authenticity of the descriptions of suffering.

The report includes a detailed account of the treatment. Nitric acid was applied ‘by the means of a small bit of sponge tied to the end of a stick’ to the whole surface of the affected breast…the object of this application was to remove the skin’.^38^ On the following day, the surgeon would slice with a scalpel, on the surface of the now exposed flesh, a series of parallel scratches, about half an inch apart, reaching from top to bottom. Then, he spread a ‘purple mucilaginous substance’ over the incisions.^39^ This substance was a paste made up from flour, chloride of zinc, and the sanguinaria root, described by Fell as ‘a root used by the North American Indians on the shores of Lake Superior, which the Indian traders told me was used by them with success in these affections’.^40^ In deriving legitimacy from the natural knowledge of the Native Americans, Fell was turning the perceived vice of his American citizenship into an exotic virtue since anti‐Americanism was rife amongst British medical men who believed that their counterparts across the Atlantic were subjected to less rigorous training and registration.^41^

The next day, the scalpel was passed again along the scratches, and the purple substance was reapplied. After a few days of this repeated exercise, narrow strips of linen rag, soaked in this purple substance, were inserted into the long parallel scores. Every day these strips of rag were renewed, and the scores were made deeper and deeper. This process killed the cancerous flesh, transforming it into ‘a woody hardness, and a deep black colour’.^42^ Once the incisions had reached the bottom of the tumour, the surgeon scored no deeper, and instead applied a ‘girdle’ around the line where the growth met living, healthy flesh. Gradually, the tumour would detach and eventually drop out of its cavity. For reasons left unexplained, none of this was conducted under chloroform.

Fell was offering this treatment as a curative intervention. He wrote long tracts defending its ability to remove the disease from the body entirely, and compared it favourably with the knife. Fell professed ‘to cure cancer by a new process, without the need of an operation’. He was committed to the curative effects of the sanguinara root – his ‘secret medicament’ – which would leave his patients ‘healed’ and ‘well’. He provided favourable figures: ‘out of every 100 cases treated [by him], not more than twenty instances occurred of a return or reappearance of the disease’.^43^ The textured accounts of pain in the Middlesex's report contrast dramatically with the cases Fell published from the same trial. One woman he reported on was ‘in excellent spirits. She says she never felt better, and all her friends say that she looks ten years younger than she did’.^44^ Another patient was described as ‘very comfortable and happy; no pain’.^45^ Yet another was quoted as saying, ‘I am able to make myself useful in my family, and to take moderate exercise without pain or tire’.^46^

However, after eight months of observation, the surgical staff at the Middlesex decided that the remedy could neither prevent nor even delay death. One patient was recorded as leaving the hospital ‘relieved indeed of her tumours, but apparently near her end from cancer of the internal organs’. The ‘cancerous energy’ was in no way dimmed by the application, and,

Under this, as under previous modes of treatment, Cancer retains its notoriously malignant character; that is to say, its capacity for spontaneous and destructive growth in its primary seat, for obstinate recurrence after what has appeared to be the most complete extirpation, and for progress, if not for reproduction, in other and, it may be, distant organs of the body.^47^

Surgeons were profoundly troubled by the likelihood of recurrence, and already at the beginning of the nineteenth century practitioners were speaking of their cancer patients in terms of years gained, rather than complete cures procured. Sufferers might be alive six months later, one year, or two, but only very rarely was this cancer‐free life permanent. In theory, too, cancers could be cured by the knife if caught very early – before they had spread to other organs and into the constitution. Surgeon Walter Hayle Walshe suggested, ‘the earlier the morbid mass is removed the stronger are the chances of ultimate recovery’.^48^ If the surgeon could intervene when the tumour was still small, still local, then they might be able to entirely prevent relapse. Italian surgeon Antonio Scarpa argued that if the ‘morbid seed’ remained ‘latent and inert’ the disease would be ‘purely *local*’ and, as a result, could be ‘susceptible of a favourable and permanent cure by extirpation’.^49^ However, this transition from phase to phase – from local to general – was unpredictable and difficult to identify from physical examination or narrative histories.

It was, therefore, somewhat unsurprising that the Middlesex surgical stuff determined that in all the cases treated by Fell ‘cancer…remains as ruthless and as unassailable as ever’.^50^ And yet, the report also concluded that the method was ‘ingenious, safe, and easy of application’.^51^ Despite its inability to arrest the malignancy of cancer or prevent recurrence, Fell's treatment was *effective*. They claimed it was ‘a clear advance upon the past’, and that this was an ‘efficacious and so manageable’ method of treating cancer.^52^ Thus, while it failed on one key count – it was not curative – it must have succeeded in some other therapeutic way. The minutes, therefore, can be read as an assessment, not of the treatment's curative value, but of its palliative efforts. And, in this assessment, they relied on their patients' words. Subjective pain narratives were thus key to constructing an expansive idea of therapeutic efficacy and to assessing palliative success.

Success was assessed along three lines: whether the treatment relieved pain, removed foetor and/or returned the body to a modicum of ‘normal’ function. Palliation in the nineteenth century meant all these things.^53^ Pain predominates in the report, which contains richly textured and varied accounts of physical suffering. The surgical staff were attuned to the fact that the treatment itself caused great distress but that the severity of the pain varied. For ‘hours together’ one woman suffered no pain, but then ‘sometimes it is only shooting and transient’.^54^ In a quotation that attests to the personality and idiosyncrasies contained in the report, another patient ‘preserved her health during all the treatment, and made light of the pain; sometimes, to show that it was not great, shaking the breast with her hand or squeezing it’.^55^ Other patients experienced ‘unusually great suffering’, and one expressed ‘herself in very strong terms as to the great severity of the pain’.^56^ Another woman described her pain as a ‘drawing sensation’, as if ‘the backbone were dragged to the breast’.^57^ These details make this source intriguing not only because of what it can tell us about how various cancer treatments were assessed by professionals, but also because it gestures towards how those treatments were experienced by the patient. For example, in one particularly harrowing quotation, a woman described the treatment as ‘dreadfully painful, as if it were pulling her heart out’.^58^ The inclusion of these emotive details suggests an interest on behalf of the surgeons in the pain and suffering experienced by their patients. This goes against prevailing interpretations of mid‐ to late‐nineteenth‐century hospital care and clinical masculinity that suggest that medical and surgical men were uninterested in the patient narrative and in the bodily and affective conditions of those they treated.^59^

## Cancer pain and cancer suffering

The surgical staff mulled over the subjectivity of physical feeling: ‘There can be no doubt that there was pain in all the cases. But pain is…far from being the same thing to all persons’.^60^ Some patients ‘were obliged to give up the treatment, and leave the hospital unrelieved’.^61^ However, in most cases, the surgical staff concluded that the pain was ‘rarely of such severity that patients, on its account, should be deprived of the eventual advantages of the treatment’.^62^ In doing so, they suggested that the treatment and its results was worth the above‐detailed suffering: pain from the treatment would limit later pain from the cancer itself. To appreciate the rationale for this equation, we need to understand quite how painful unchecked cancer could be. We know from various sources – medical tracts, newspaper articles, patient accounts – that late‐stage malignancy was almost unbearable in the suffering it inflicted. Patients described the pain by analogy to ‘the prodding of knives, incision with lancets’.^63^ An advert posted in *The Leeds Mercury* in 1844 included a testimony from Elizabeth Crowther who had been suffering ‘under extreme pain’ from cancer of the breast.^64^ Newspapers, periodicals and medical dictionaries layered a frank and open discourse about the peculiar pain and distress that accompanied dying from cancer – obituaries often included lengthy and candid descriptions of protracted suffering. Diagnosed with cancer, few in the nineteenth century would be under any illusions as to their eventual fate.

However, pain was not the only feature of the cancer experience. Physical suffering was almost invariably accompanied by foetid flesh, and patients experienced distress and anxiety associated with the ‘disgusting’ side effects of the diseased body.^65^ Histories and sociologies of palliative care tend to focus on pain to the exclusion of the other effects of dying.^66^ But in this case, relieving the other effects was just as, if not more, crucial than the numbing of physical pain. Left to its own devices, the cancerous body was increasingly abject, ‘the fact of a sanies of fetid odor and peculiarly acrid qualities being more or less abundantly thrown out by the disorganised surface’.^67^ This description by surgeon Walter Hayle Walshe was in part metaphorical, alluding to the anxieties provoked by the diseased state. The surface was ‘disorganised’ – no longer in its proper order, no longer aligning with what we expect and can predict. He reflected on the benefits conferred by dying from a cancer of the internal organs rather than from a tumour on the skin's surface – they were saved from the ‘painful and disgusting’ death that attends cancer ‘when allowed to proceed in its natural course to a fatal termination’.^68^

Thus, palliative efforts in the Middlesex Hospital worked to re‐control the body's boundaries and return integrity to its surface. Fell's treatment involved the removal of diseased and foetid flesh. According to the minutes, this was always ‘succeeded by a healthy granulating and cicatrising surface’.^69^ Cancerous tumours ulcerated and seeped noxious fluids and Fell's treatment repaired the body's boundaries. This was exemplified by the case of one woman whose breast cancer emitted such a foul smell that even Burnett's solution, ‘employed plentifully about the bed and outside the dressing’, could not correct it.^70^ And yet, Fell's application immediately destroyed the ‘offensive smell’ which had once seeped out beyond her bed. This ability was one of the only aspects of the treatment that Philip Henry Gosse praised. He wrote that the application,

…Had evidently a caustic power…for the part destroyed had no tendency to decomposition; it was brought to a woody hardness, and a deep black colour, without the least odour. It was the one merciful mitigation of her sufferings, that, all the time she was under Dr F., not the slightest offensive odour was perceptible from the disease.^71^

One patient wrote to a nurse at the Middlesex a letter ‘full of expressions of gratitude’, saying that her ‘breast is healed nicely’.^72^ Second to a cure, the ‘healing’ of open sores was the priority for both doctor and patient. Because Fell's treatment sealed off the ulcerating tumour, it served to reconstitute the body, and partially return the patient to their unviolated, pre‐cancerous form.

It is important to remember that these patients at the Middlesex were mostly drawn from the surrounding areas of intense urban poverty. Close quarters living likely made someone suffering with a seeping, offensive tumour unwelcome. Moreover, while cancerous ulcerations and suppurations were no doubt unpleasant, they also prevented sufferers from performing day‐to‐day duties and pleasures. People sought hospital care and surgical consult when they could no longer remain at home and in their communities, either because their foetid flesh made them unsociable or because they became a burden on their household economies. According to the surgical report, one ‘great advantage’ that attended Fell's mode of treatment was that, unlike after surgery, the patients were not confined to bed or to the house, but, ‘on the contrary, they are able to obtain the benefit of exercise in the open air’.^73^ They invoked a holistic understanding of health and appreciated that the treatment might have many and various outcomes, not just effect a cure. In their write‐up, they recorded ‘as to the General Health of the patients: In not a few instances it has been positively improved under the treatment’.^74^

Other accounts of surgical palliation recorded the ability of the intervention to return patient's bodies to relative, albeit temporary, function. Mid‐century surgeon, James Young Simpson, wrote about the recovery of a patient with cervical cancer: ‘After the excision of the diseased structures the lumbar pains and local watery and haemorrhage discharges entirely ceased…The patient rallied so speedily in health and strength, that within a fortnight she was able to be taken into the garden’.^75^ This mundane detail about the woman's leisure possibilities emphasised the very human relationship between patient and practitioner. In a similar case involving a different surgeon, J. Argyll Robertson from University College, London detailed the return to health of one of his patients after the removal of a cancerous eye. Robertson quotes his patient as being ‘able to do a good day's work yet at his trade of weaving’.^76^ Palliation meant not only a reduction in pain, but an ability to return to daily life and work. Moreover, this focus on people's leisure and labour suggests efforts to put across an image of comprehensive compassion.

These surgeons portrayed sympathy as a key – if not the only – motivation for allowing Fell to conduct his trial. They were invested in the alleviation of suffering and they incorporated the thoughts and feelings of their patients into their clinical texts. They refer to their patients’ ‘agitation of mind’, ‘acute distress’ and one individual's ‘feeling of intense relief’. They comment on their physical state – ‘her sufferings are great, and her strength is much exhausted’ – as well as their emotional condition – ‘The patient, originally dejected, and made still worse by repeated bilious attacks, to which she was subject, was grieved beyond measure at the ill‐success of the treatment’.^77^ They also presented themselves as emotionally astute, often reading untold suffering into their patients’ silences. One woman complained of great pain, and the surgeon recorded that ‘her looks confirmed what she said’.^78^ Another was apparently silent on the subject, but ‘it was obvious…that she was greatly harassed by the mode of treatment adopted’. Some, however, remained mysteries: ‘No one could tell what she suffered’. These affective accounts not only disrupt the historiographical notion that, as the nineteenth century progressed, the patient narrative disappeared from clinical record and process, but also disrupts our expectations of surgical dispassion.^79^ In their edited volume, *Science Incarnate*, Steven Shapin and Christopher Lawrence queried the pervasive assumption that ‘Science is made by people without bodies – by purely mental entities without passions, desires or genders’.^80^ On the contrary, they insisted that ‘scientific knowledge was made by embodied human beings’. Crucially, too, the human beings examined here were men ‘whose masculinity was not entirely accidental to their vocation’.^81^

Not only were these surgical practitioners men with bodies, they were men with emotions.^82^ Historian Michael Brown has written about an early nineteenth century ‘culture of sensibility’ which evolved into a version of ‘Romantic sensibility’ identified by its ‘veneration of honest, heartfelt feeling and its particular emphasis upon the sympathetic treatment of women and children’.^83^ While it is generally thought to have declined after the 1830s, this case study demonstrates that remnants appear in later Victorian surgical culture. For the surgical staff at the mid‐century Middlesex, their affective descriptions of suffering were part of the construction of a persona that positioned care and compassion as crucial and useful parts of their professional activity and identity. Due to surgeons’ close and recent association with the pain, misery and likely mortality of operations without anaesthesia, they were under particular pressure to cultivate an image of considered kindness and clinical efficacy. They presented their surgical duty as to alleviate both physical pain and emotional distress. Cancer's chronic and incurable status – and its capacity to make the body abject – made it unusually useful in the construction of a well‐rounded professional and masculine identity.

According to the surgical staff, one ‘very *important advantage*’ to Fell's application was that it expanded the number of cancer sufferers who might be treated in the hospital: it admitted ‘to the benefit of treatment an entirely new class of those who suffer from cancer – *a class* hitherto almost *universally* abandoned, at least in *England*’.^84^ This ‘class’ consisted of those whose cancers had advanced to such a late stage that any surgical intervention was deemed by most to be far too dangerous. Most of the patients treated by Fell's application in the Middlesex were of this ‘class’ – those who were beyond all other hope. This status also made possible their inclusion in the trial at all; because all else had failed this was their last chance at relief. Nineteenth‐century cancer discourse is replete with this sort of melodramatic distress. Physicians and surgeons derived social and professional capital from lamentations and doubt over cancer. Malignancy allowed them to articulate a coherent image and identity, grounded in their feelings for patients’ suffering, and predicated on values of humanity and humility. And, to a degree, it worked. A writer reporting on Fell's trial wrote ‘There is no class of men more thoughtfully and *actively* benevolent than those belonging to the medical profession’.^85^ A representation quite unlike ‘the dominant code of Victorian manliness’ that emphasised self‐control and stoicism.^86^

## Emily Bowes Gosse

In 1857, the spiritual writer and painter Emily Bowes Gosse, wife of the Victorian naturalist and Plymouth Brethren Philip Henry Gosse, died from breast cancer. Her husband wrote a remarkable text on her experience of treatment, subsequent decline and death. It provides a moving and harrowing account of her final days and of the suffering that accompanied cancer in the nineteenth century. Alarmed at the discovery of a lump in her breast, Emily showed it to her ‘tried friend, Miss Stacey of Tottenham’ who immediately took her to visit a local doctor.^87^ Diagnosing her with cancer, Dr Laseron recommended ‘instant excision’.^88^ But, Philip's relative had heard of an American ‘who professed to cure cancer by a new process, without the need of an operation’.^89^ Fell promised a complete cure, ‘without recourse to the knife’, and showed the Gosses ‘photographs of many patients in different stages of cure, many large tumours preserved in spirit’.^90^ He assured the anxious couple that out of every 100 cases treated with his application, no more than twenty saw a recurrence of cancer. Here Philip interrupts his narrative retelling of Emily's treatment to caution the reader with a melancholic reflection, ‘The treatment resorted to did really (as I believe was the case) aggravate my beloved's sufferings and hasten her death’.^91^

From the 12 May to the end of August 1856, Fell applied two or three kinds of ointment to Emily's breast, which ‘produced such intense aching and “drawing” pain in the tumour, that altogether it was a time of much suffering’.^92^ In October, Fell advised the removal of the tumour because the ointment had failed to produce any great change in the breast's appearance.^93^ Philip described the process of extraction in detail, and it conformed exactly to the description provided in the surgical staff's minutes. Philip wrote,

The effect of this application was very distressing. In about an hour after its renewal every morning, the breast began to be the seat of an aching, piercing pain, under which my beloved sufferer was fain to wander up and down her narrow room, leaning now and then her head upon the mantel‐piece or against the wall, unable from the agony to lie, sit, or stand.^94^

This immediate distress continued for several hours, after which the intensity diminished. However, ‘abatement of suffering…was the most she could look for; suffering never *ceased* from the beginning of the operation, till her spirit was freed from the worn‐out body’.^95^ Throughout, Philip insisted that Emily behaved according to feminine and evangelical codes of gentility and stoicism: ‘she resigned herself to the new torture with calm submission to her Father's will. Indeed, amidst all the sights and moans wrung from her in the course of this sore affliction, I never heard her utter a single murmuring word; not an expression, not a look, that intimated a doubt of the loving‐kindness of the Lord’.^96^

When the incisions had reached the depth of about an inch and a quarter, Fell announced that he had reached the bottom of the cancer. He applied a ‘girdle’ around the line where the killed tumour joined the living flesh. The aim now was to provoke the tumour to gradually detach from its body, ‘like a stone dropped out of a basin’.^97^ Two weeks later, on Sunday, the 23^rd^ of November, and to the couple's ‘delight’, the ‘great insensible tumour fell out of its cavity…and the breast was relieved of its load – the dead body that it had so long carried about’.^98^ Despite initial relief, the Gosses were soon disappointed. Further tumours were found deeper in the breast and Fell advised further painful treatment.

Almost a month later, after the discovery of yet more tumours buried deep in Emily's chest, she asked Fell, ‘But how do you account for this spreading of the disease beyond the part you have all along been dealing with?’ Fell responded, ‘Oh, ‘tis in your blood’.^99^ Emily, according to Philip, ‘calmly took her leave’ and returned home. Together, they decided not to undergo any further treatment. Philip asked his readership, ‘what is the use of a merely local treatment of a disease which is seated in the blood?’.^100^ Likely Philip was aware that this question resonated with the contemporary debate over the utility of surgery in cases of cancer (described above). Emily appeared to be one such case that had been caught too late.

Philip clearly resented Fell and his dishonesty. He insisted that throughout the process Fell had assured the couple that ‘cancer was a local, and not a constitutional disease’.^101^ Therefore, Fell's claim to the contrary, took the Gosses ‘by surprise’.^102^ He called Fell's application ‘hopeless’, and wrote about how ‘the cup [had been] dashed from our lips’.^103^ Emily's decision to discontinue Fell's treatment presented the couple with a dilemma. Should they seek orthodox surgical consult or go elsewhere for homeopathic remedies? Philip reflected on the decision: ‘It is a solemn position to be placed when one is called to choose between rival systems of medicine, with the feeling that life and death are in the balance’.^104^ After some undescribed and ineffective homeopathic treatment, Emily began a rapid decline. In the January before her death, she ‘suffered very much from sleeplessness’ and underwent ‘fits of exceeding depression’.^105^ At night, she experienced ‘the most painful restlessness’, and an inability to remain for more than a moment in one place or position.^106^ She lost the use of her left arm, and her shoulder ached. These localised pains were accompanied by ‘shifting rheumatoid pains in the body’, fever and chills, paroxysms of coughing, and a general bodily weakness.^107^ She lost the ability to speak or swallow.^108^ Gosse ‘complained of the dullness of her spirit’ and ‘she was unable to read more than a verse or two at a time; unable even to bear that one should read to her, if the subject required the least thought’.^109^ Finally,

She could not be raised in the bed without strong pains in the loins; her nights, from the terrible paroxysms of coughing, were frequently wretched; the power of using the lower limbs as well as the upper, on the left side, was almost wholly lost; bed‐sored appeared; breathing was performed with increased difficulty.^110^

She died on 10 February 1857.

This text is richly descriptive and intensely emotional. Philip paints a vivid picture of his wife's suffering and articulates his own wretched disappointment at the ill‐success of her treatment. This runs counter not only to our assumptions about Philip Henry Gosse as an individual, but also to what we expect of nineteenth‐century scientific men as a collective. Edmund Gosse famously describes his father as unfeeling; however, as their biographer Anne Thwaite argues, he was a tenderly devoted father and husband and his marriage to Emily was a happy one.^111^ While Phillip suggests that Emily conformed to some gendered and classed norms in her suffering, he does not set himself – a man – in opposition.^112^ He does not align himself with conventional narratives of Victorian masculinity. Instead, he repeatedly calls her his ‘beloved’ and references his own frantic emotional state, describing himself at various points as delighted, distressed, anxious and disappointed. The account does not present Philip as possessing some of the prevailing masculine character traits. He is neither a ‘man of action’ or a ‘cool rationalist’, but an emotional husband sharing in his wife's suffering.^113^

However, this was not just a romantic narrative – and Gosse's sympathy for his wife was not mawkish, sentimental or feminine. Rather, he frequently restates his scientific identity. He records the treatment's ‘antiseptic property’, interjecting his technical knowledge into an otherwise affective and affecting text. Despite being full of emotion, the account is also dense with detail and analysis. He describes the process fully and gives an intricate account of her symptoms. Even during his wife's final days, he retains the observational aptitude of a natural scientist. His discussion of whether cancer is a constitutional or local disease references a contemporary medical debate and demonstrates an interest in the pathological processes behind his wife's suffering. In this way, Philip integrates the affective and the scientific to forge a distinct identity – one that blends multiple personae. Like the surgical staff at the Middlesex Hospital, Philip was constructing a compassionate professional image in which scientific acumen was entirely compatible with spousal love and emotional intimacy.

## Conclusion

This article has used a case study – Dr J. Weldon Fell's new treatment for cancer – to explore expressions of masculine identity in accounts of physical and emotional suffering. I argued that the surgical staff of the Middlesex Hospital used their patients’ descriptions of agony, disgust and incapacity to assess the therapeutic efficacy of Fell's treatment. This was particularly important because the surgical staff believed Fell's intervention to be palliative rather than curative and so could not rely on observable, clinical signs of therapeutic success or failure. Instead, doctors had to depend on a complex calculus of pain and suffering, and consider an expansive and holistic interpretation of health and wellbeing to decide whether to incorporate Fell's treatment into their clinical arsenal. I also suggested that both the hospital's surgical staff and Philip Henry Gosse used their affective narratives of female suffering to construct an image of caring and invested professional men and, in doing so, subverted or complicated traditional ideas of Victorian masculinity and scientific detachment.

We can interpret these efforts of self‐presentation as performative or cynical, as market‐driven or instrumentalist. However, the context of each text suggests otherwise. The hospital case notes were not intended to be part of public debate or popular lexicon, and it is difficult to imagine what direct personal gains Philip Henry Gosse might have made from expressions of spousal compassion. Indeed, it is likely that his intention behind publication was to discredit Fell and his treatment. Thus, I would like to suggest that while deliberate and instrumental ‘uses’ of affective rhetoric and compassion played a part in both texts, we might also take a degree of expressed distress and sympathy at face‐value. Philip was likely profoundly distressed at the decline and death of his wife, while also appreciating that his affective account would resonate with a particular version of nineteenth‐century masculinity. It is possible the Middlesex surgeons cared deeply about their patients, valued their voices and were moved to try unorthodox methods to relieve their suffering; however, it is also possible that they recognised the social and professional utility of framing their practice in such terms.

Many of the concerns presented in this article still preoccupy twenty‐first‐century doctors and patients. A cure for cancer continues to elude the medical profession and today more than half of us will live with or die from cancer. While the dramatic manifestations of advanced and untreated malignancy – decayed flesh, putrid odour, ulcerating tumours – are rarely seen in industrialised countries, many patients today must decide whether to accept a therapy that induces harsh side effects but provides only temporary relief.^114^ Indeed, the language we use to talk about cancer today – the language of remission – obscures the essentially palliative nature of many of the treatments deployed. Finally, while foetid tumours might be rare now, the dying cancer patient's body remains abject. Frail, pained and distorted – Gosse's description of his wife's body and her suffering will likely be recognisable to anyone who has sat with a cancer death in the twenty‐first century. We might express our feelings in different terms, but his account retains a profoundly emotive quality and gestures towards the disappointment and despair that continues to accompany the disease today.

## Notes

1. Alexander Shaw, Charles H. Moore, Campbell de Morgan, and Mitchell Henry, *Report of the Surgical Staff of the Middlesex Hospital, to the Weekly Board and Governors, Upon the Treatment of Cancerous Diseases in the Hospital, on the Plan Introduced by Dr. Fell* (London: John Churchill, 1857), p. 2.

2. Philip Henry Gosse, *A Memorial of the Last Days on Earth of Emily Gosse* (London: J. Nisbet & Co., 1857).

3. ‘Reviewed Work: *Report of the Surgical Staff of the Middlesex Hospital to the Weekly Board and Governors upon the Treatment of Cancerous Diseases in the Hospital on the Plan Introduced by Dr Fell’, The British Medical Journal* 2 (1857), pp. 888–889, here p. 888.

4. L. R. Croft, ‘Edmund Gosse and the “New and Fantastic Cure” for Breast Cancer’, *Medical History* 38 (1994), pp. 143–59, here p. 144.

5. Gosse, *A Memorial of the Last Days on Earth of Emily Gosse*, p. ix.

6. John Tosh, ‘What Should Historians Do with Masculinity? Reflections on Nineteenth‐Century Britain’, *History Workshop Journal* 38 (1994), pp. 179–202, here p. 183.

7. Stefan Collini, ‘Having Emotions the Manly Way’, *Times Literary Supplement*, (June 4, 1999), here p. 6.

8. See Lindsey Fitzharris, *The Butchering Art: Joseph Lister's Quest to Transform the Grisly World of Victorian Medicine* (London: Penguin, 2017). While we might have attained a more nuanced understanding of nineteenth‐century masculinity broadly defined, we have done less well in complicating our narratives of nineteenth‐century scientists and doctors.

9. L. R. Croft, ‘Gosse, Philip Henry (1810–1888), Zoologist and Religious Writer’, *Oxford Dictionary of National Biography*, 1 December 2017.

10. Edmund Gosse, *Father and Son*, ed. Michael Newton, (Oxford: Oxford University Press, 2004).

11. Aileen Fyfe, ‘Conscientious Workmen or Booksellers’ Hacks? The Professional Identities of Science Writers in the Mid‐Nineteenth Century’, *Isis* 96 (2005), pp. 192–223, here p. 193.

12. Gosse, *Father and Son*, p. xvii.

13. An exception is, Croft, ‘Edmund Gosse and the “New and Fantastic Cure” for Breast Cancer’. However, this article focuses more on the public reception of Fell and his questionable status as professional physician, rather than the emotional dynamics and the gendered nature of the doctor‐patient relationship.

14. Scholars have conceptualised the nineteenth century as the ‘epidemic century’, with infectious diseases occupying the forefront of historical investigation. This periodisation is most clearly articulated by Abdel Omran, ‘The Epidemiological Transition: A Theory of the Epidemiology of Population Change’, *The Millbank Quarterly* 83 (1971), pp. 731–57.

15. Ornella Moscucci, *Gender and Cancer in England, 1860–1948* (London: Palgave Macmillan, 2016).

16. Tammy Duerden Comeau, ‘Gender Ideology and Disease Theory: Classifying Cancer in Nineteenth Century Britain’, *Journal of Historical Sociology* 20 (2007), pp. 158–81.

17. Heather Ellis, *Masculinity and Science in Britain, 1831–1918* (London: Palgrave Macmillan, 2017), p. 2.

18. Jan Golinski, ‘Humphry Davy's Sexual Chemistry’, *Configurations* 7 (1999), pp. 15–41, here p. 15.

19. Ellis, *Masculinity and Science in Britain, 1831–1918*, p. 3.

20. Jan Golinski, ‘The Care of the Self and the Masculine Birth of Science’, *History of Science* 40 (2002), pp. 125–45, here pp. 126–7.

21. Golinski, ‘The Care of the Self and the Masculine Birth of Science’, p. 128.

22. Jan Golinsky, ‘Humphry Davy: The Experimental Self’, *Eighteenth Century Studies* 45 (2011), pp. 15–28, here p. 24.

23. Michael Brown, ‘The Compassionate Surgeon: Lessons from the Past’, *The Bulletin of The Royal College of Surgeons* 98 (2016), pp. 28–29.

24. Lynda Payne, *With Words and Knives: Learning Medical Dispassion in Early Modern England* (London: Ashgate, 2007).

25. See: Fitzharris, *The Butchering Art* (London: Penguin UK, 2017); and, Ornella Mosuccci, *The Science of Woman: Gynaecology and Gender in England, 1800–1929* (Cambridge: Cambridge University Press, 1993).

26. Joanna Bourke, ‘Pain, Sympathy and the Medical Encounter Between the Mid‐Eighteenth and the Mid‐Twentieth Centuries’, *Historical Research* 85 (199), pp. 430–52; Joanna Bourke, *Pain and the Politics of Sympathy: Historical Reflections 1760s to 1960s* (Utrecht: Universiteit Utrecht, 2011) and Michael Brown, ‘Redeeming Mr. Sawbone: Compassion and Care in the Cultures of Nineteenth‐Century Surgery’, *Journal of Compassionate Health Care* 4 (2017).

27. I borrow the phrase a ‘calculus of suffering’ from Martin S. Pernick, *A Calculus of Suffering: Pain, Professionalism, and Anesthesia in Nineteenth‐century America* (New York: Columbia University Press, 1985).

28. UCLH Archive, London, ‘Weekly Board Meeting’, Middlesex Hospital Minutes, 29 November 1791.

29. UCLH Archive, London, ‘Weekly Board Meeting’, Middlesex Hospital Minutes, 29 November 1791.

30. UCLH Archive, London, ‘Weekly Board Meeting’, Middlesex Hospital Minutes, January 10, 1792.

31. UCLH Archive, London, ‘Weekly Board Meeting’, Middlesex Hospital Minutes, January 10, 1792.

32. UCLH Archive, London, ‘Weekly Board Meeting’, Middlesex Hospital Minutes, January 10, 1792.

33. UCLH Archive, London, ‘Weekly Board Meeting’, Middlesex Hospital Minutes, January 10, 1792.

34. UCLH Archive, London, ‘Weekly Board Meeting’, Middlesex Hospital Minutes, January 10, 1792.

35. Burney was an English gentlewoman who lived in France at the beginning of the nineteenth century who recorded a vivid account of her own pre‐anaesthesia mastectomy. J. E. Epstein has looked at Burney's narrative, exploring issues of gender and maternity. (J. E. Epstein, ‘Writing the Unspeakable: Fanny Burney's Mastectomy and the Fictive Body', *Representations* 16 (1986), pp. 131–66.)

36. Shaw et al*, Report of the Surgical Staff of the Middlesex Hospital*, p. 3.

37. Mrs. Valentine Bartholomew wrote, ‘His heart seems filled with sympathy to overflowing for the sufferers of his race’. Valentine Bartholomew, ‘Dr Fell Versus Cancer’, *The Ladies’ Cabinet*, 1 November 1856, p. 268.

38. The use of corrosive substances to remove breast tumours is part of a long tradition. See Carroll Smith Rosenberg and Charles Rosenberg, ‘The Female Animal: Medical and Biological Views of Woman and Her Role in Nineteenth‐Century America’, *Journal of American History* 60 (1973), pp. 332–356; Ornella Moscucci, *The Science of Woman: Gynecology and Gender in England, 1800–1929* (Cambridge: Cambridge University Press, 1990).

39. Shaw et al*, Report of the Surgical Staff of the Middlesex Hospital*, p. 5.

40. J. Weldon Fell, *A Treatise on Cancer, and its Treatment*, (London: John Churchill, 1857), pp. 56–7.

41. Broadly speaking, medical training and practice was less regulated in the United States than in Britain. See George Weiss, ‘Medical Directories and Medical Specialization in France, Britain, and the United States’, *Bulletin of the History of Medicine* 71 (1997), pp. 29–31.

42. Gosse, *A Memorial of the Last Days on Earth of Emily Gosse*, pp. 31–2.

43. Fell, *A Treatise on Cancer, and its Treatment*, p. 56.

44. Fell, *A Treatise on Cancer, and its Treatment*, p. 68.

45. Fell, *A Treatise on Cancer, and its Treatment*, p. 71.

46. Fell, *A Treatise on Cancer, and its Treatment*, p. 77.

47. Shaw et al*, Report of the Surgical Staff of the Middlesex Hospital*, p. 35.

48. Walter Hayle Walshe, *The Anatomy, Physiology, Pathology, and Treatment of Cancer* (Boston: [Publisher?], 1844), p. 151.

49. Antonio Scarpa, *Remarks and Practical Results of Observation on Scirrhus and Cancer*, trans. James Briggs, (London, 1822), p. 29.

50. Shaw et al*, Report of the Surgical Staff of the Middlesex Hospital*, p. 50.

51. Shaw et al, *Report of the Surgical Staff of the Middlesex Hospital*, p. 8.

52. Shaw et al, *Report of the Surgical Staff of the Middlesex Hospital*, p. 42.

53. ‘Palliative’, ‘palliation’, and ‘palliate’ were used throughout the nineteenth century and before, and I borrow my actors’ phrasing in describing their theory and practice.

54. Shaw et al*, Report of the Surgical Staff of the Middlesex Hospital*, p. 89.

55. Shaw et al, *Report of the Surgical Staff of the Middlesex Hospital*, p. 77.

56. Shaw et al, *Report of the Surgical Staff of the Middlesex Hospital*, p. 87.

57. Shaw et al, *Report of the Surgical Staff of the Middlesex Hospital*, p. 63.

58. Shaw et al, *Report of the Surgical Staff of the Middlesex Hospital*, p. 63.

59. Nicholas D. Jewson, ‘The Disappearance of the Sick‐Man from Medical Cosmology, 1770–1870’, *Sociology* 10 (1976), pp. 225–244.

60. Jewson, ‘The Disappearance of the Sick‐Man from Medical Cosmology, 1770–1870’, p. 38.

61. Jewson, ‘The Disappearance of the Sick‐Man from Medical Cosmology, 1770–1870’, p. 39.

62. Jewson, ‘The Disappearance of the Sick‐Man from Medical Cosmology, 1770–1870’, p. 42.

63. Hayle Walshe, *The Anatomy, Physiology, Pathology, and Treatment of Cancer*, p. 136.

64. ‘Advertisements & Notices’, *The Leeds Mercury*, 24 February 1844.

65. The idea that the suffering produced by cancer was not limited to pain has been explored by various historians of medicine, including Patrice Pinell, *The Fight Against Cancer: France 1890–1940* (London: Routledge, 2002); Ilana Löwy, *Woman's Diseases* (Cambridge: Cambridge University Press, 2011); Robert Aronowitz, *Unnatural History: Breast Cancer and American Society* (Cambridge: Cambridge University Press, 2007).

66. Julia Lawton, *The Dying Process: Patients’ Experiences of Palliative Care* (London: Routledge, 2000).

67. Walter Hayle Walshe, *The Nature and Treatment of Cancer* (London, 1844), p. 124.

68. Ibid, pp. 245–246.

69. Shaw et al*, Report of the Surgical Staff of the Middlesex Hospital*, p. 10.

70. Burnett's Solution was a popular disinfectant. Shaw et al, *Report of the Surgical Staff of the Middlesex Hospital*, p. 67.

71. Gosse, *A Memorial of the Last Days on Earth of Emily Gosse*, pp. 31–32.

72. Shaw et al*, Report of the Surgical Staff of the Middlesex Hospital*, p. 72.

73. Shaw et al*, Report of the Surgical Staff of the Middlesex Hospital*, p. 10.

74. Shaw et al, *Report of the Surgical Staff of the Middlesex Hospital*, p. 33.

75. James Young Simpson, *Cases of Excision of the Cervix Uteri for Carcinomatous Disease* (Dublin, 1846), p. 7.

76. J. Argyll Robertson, *Excision of the Eyeball in Cases of Melanosis, Medullary Carcinoma, and Carcinoma: With Remarks* (London, 1844), p. 8.

77. Shaw et al*, Report of the Surgical Staff of the Middlesex Hospital*, p. 94.

78. Shaw et al, *Report of the Surgical Staff of the Middlesex Hospital*, p. 79.

79. Nicholas D. Jewson, ‘The Disappearance of the Sick‐Man from Medical Cosmology, 1770–1870’, *Sociology* 10 (1976), pp. 225–244.

80. Christopher Lawrence and Steven Shapin, (eds.), *Science Incarnate: Historical Embodiments of Natural Knowledge* (Chicago: Chicago University Press, 1998).

81. Golinski, ‘The Care of the Self and the Masculine Birth of Science’, p. 128.

82. ‘The sympathy that physicians possessed was neither feminine nor apeish: it was masculine’, Bourke, ‘Pain, Sympathy and the Medical Encounter Between the Mid‐Eighteenth and the Mid‐Twentieth Centuries’, p. 440.

83. Brown, ‘Redeeming Mr. Sawbone: Compassion and Care in the Cultures of Nineteenth‐Century Surgery’.

84. Shaw et al*, Report of the Surgical Staff of the Middlesex Hospital*, p. 62.

85. Bartholomew, ‘Dr Fell Versus Cancer’, p. 268.

86. John Tosh, ‘What Should Historians Do with Masculinity? Reflections on Nineteenth‐Century Britain’, *History Workshop Journal* 38 (1994), pp. 179–202, here p. 183.

87. Gosse, *A Memorial of the Last Days on Earth of Emily Gosse*, p. 5.

88. Gosse, *A Memorial of the Last Days on Earth of Emily Gosse*, p. 5.

89. Gosse, *A Memorial of the Last Days on Earth of Emily Gosse*, p. 6.

90. Gosse, *A Memorial of the Last Days on Earth of Emily Gosse*, p. 10.

91. Gosse, *A Memorial of the Last Days on Earth of Emily Gosse*, p. 10.

92. Gosse, *A Memorial of the Last Days on Earth of Emily Gosse*, p. 19.

93. Gosse, *A Memorial of the Last Days on Earth of Emily Gosse*, p. 19.

94. Gosse, *A Memorial of the Last Days on Earth of Emily Gosse*, pp. 29–30.

95. Gosse, *A Memorial of the Last Days on Earth of Emily Gosse*, p. 30.

96. Gosse, *A Memorial of the Last Days on Earth of Emily Gosse*, pp. 35–36.

97. Gosse, *A Memorial of the Last Days on Earth of Emily Gosse*, p. 33.

98. Gosse, *A Memorial of the Last Days on Earth of Emily Gosse*, p. 33.

99. Gosse, *A Memorial of the Last Days on Earth of Emily Gosse*, p. 45.

100. Gosse, *A Memorial of the Last Days on Earth of Emily Gosse*, pp. 45–46.

101. Gosse, *A Memorial of the Last Days on Earth of Emily Gosse*, p. 45.

102. Gosse, *A Memorial of the Last Days on Earth of Emily Gosse*, p. 45.

103. Gosse, *A Memorial of the Last Days on Earth of Emily Gosse*, p. 35.

104. Gosse, *A Memorial of the Last Days on Earth of Emily Gosse*, p. 47.

105. Gosse, *A Memorial of the Last Days on Earth of Emily Gosse*, p. 48.

106. Gosse, *A Memorial of the Last Days on Earth of Emily Gosse*, p. 48.

107. Gosse, *A Memorial of the Last Days on Earth of Emily Gosse*, p. 49.

108. Gosse, *A Memorial of the Last Days on Earth of Emily Gosse*, p. 61.

109. Gosse, *A Memorial of the Last Days on Earth of Emily Gosse*, p. 40.

110. Gosse, *A Memorial of the Last Days on Earth of Emily Gosse*, p. 61.

111. Ann Thwaite, *Glimpses of the Wonderful: The Life of Philip Henry Gosse, 1810–1888* (London: Faber & Faber, 2002), p. xix.

112. Joan Scott writes, ‘The principle of masculinity rests on the necessary repression of feminine aspects’. (Joan Wallach Scott, *Gender and the Politics of History* (New York, 1988), p. 38.) However, John Tosh has argued that masculinity in nineteenth‐century Britain was, ‘only secondarily about men's relations with women’. (Tosh, ‘What Should Historians do with Masculinity?’, p. 183.)

113. Golinski, ‘Humphry Davy's Sexual Chemistry’, *Configurations* 7 (1999), p. 15.

114. These consequences of cancer continue to be present in low‐ and middle‐income countries. See, Julie Livingston, *Improvising Medicine: An African Oncology Ward in an Emerging Cancer Epidemic* (Duke University Press, 2011).

